# Antibiotic Prescribing in Outpatient Settings: Rural Patients Are More Likely to Receive Fluoroquinolones and Longer Antibiotic Courses

**DOI:** 10.3390/antibiotics12020224

**Published:** 2023-01-20

**Authors:** Haley J. Appaneal, Aisling R. Caffrey, Vrishali Lopes, David Dosa, Kerry L. LaPlante

**Affiliations:** 1 Infectious Diseases Research Program, Providence Veterans Affairs Medical Center, Providence, RI 02908, USA; 2 Center of Innovation in Long-Term Support Services, Providence Veterans Affairs Medical Center, Providence, RI 02908, USA; 3 College of Pharmacy, University of Rhode Island, Kingston, RI 02881, USA; 4 School of Public Health, Brown University, Providence, RI 02903, USA; 5 Warren Alpert Medical School, Brown University, Providence, RI 02903, USA

**Keywords:** rural, antibiotics, fluoroquinolones, duration, suboptimal antibiotic use

## Abstract

Suboptimal antibiotic prescribing may be more common in patients living in rural versus urban areas due to various factors such as decreased access to care and diagnostic testing equipment. Prior work demonstrated a rural health disparity of overprescribing antibiotics and longer durations of antibiotic therapy in the United States; however, large-scale evaluations are limited. We evaluated the association of rural residence with suboptimal outpatient antibiotic use in the national Veterans Affairs (VA) system. Outpatient antibiotic dispensing was assessed for the veterans diagnosed with an upper respiratory tract infection (URI), pneumonia (PNA), urinary tract infection (UTI), or skin and soft tissue infection (SSTI) in 2010–2020. Rural–urban status was determined using rural–urban commuting area codes. Suboptimal antibiotic use was defined as (1) outpatient fluoroquinolone dispensing and (2) longer antibiotic courses (>ten days). Geographic variation in suboptimal antibiotic use was mapped. Time trends in suboptimal antibiotic use were assessed with Joinpoint regression. While controlling for confounding, the association of rurality and suboptimal antibiotic use was assessed with generalized linear mixed models with a binary distribution and logit link, accounting for clustering by region and year. Of the 1,405,642 veterans diagnosed with a URI, PNA, UTI, or SSTI and dispensed an outpatient antibiotic, 22.8% were rural-residing. In 2010–2020, in the rural- and urban-residing veterans, the proportion of dispensed fluoroquinolones declined by 9.9% and 10.6% per year, respectively. The rural-residing veterans were more likely to be prescribed fluoroquinolones (19.0% vs. 17.5%; adjusted odds ratio (aOR), 1.03; 95% confidence interval (CI), 1.02–1.04) and longer antibiotic courses (53.8% vs. 48.5%; aOR, 1.19, 95% CI, 1.18–1.20) than the urban-residing veterans. Among a large national cohort of veterans diagnosed with URIs, PNA, UTIs, and SSTIs, fluoroquinolone use and longer antibiotic courses were disproportionally more common among rural- as compared to urban-residing veterans. Outpatient antibiotic prescribing must be improved, particularly for rural-residing patients. There are many possible solutions, of which antibiotic stewardship interventions are but one.

## 1. Introduction

Appropriate antibiotic use is an important public health priority in all healthcare settings. The majority of antibiotic expenditures (>60%) and consumption (80–90%) occur in outpatients [1]. Each year in the United States (US), over 266 million courses of antibiotics are prescribed in outpatient settings [2]. It is estimated that up to 50% of all outpatient antibiotic use is inappropriate, including unnecessary use and inappropriate drug selection, dose, and/or duration [3,4]. Previous work suggests a rural health disparity in overprescribing antibiotics and using longer antibiotic durations in the US [4,5,6,7,8]. Inappropriate antibiotic prescribing is highly prevalent in rural areas, and rurality of the healthcare practice is an independent predictor of potentially inappropriate antimicrobial prescribing [9,10,11]. Primary care patients in Canada who received care at a rural versus urban/suburban practice had a higher odds (odds ratio, 1.47; confidence interval, 1.17–1.84) of receiving an antimicrobial prescription for a viral infection [11]. However, there has only been one large-scale evaluation of differences in inappropriate outpatient antibiotic prescribing between rural- and urban-residing patients [6].

Fluoroquinolone use and long courses of therapy are two important process metrics targeted by outpatient antibiotic stewardship programs. Despite their utility as a broad-spectrum option for urinary tract infections (UTIs), cellulitis, and upper respiratory infections, oral fluoroquinolones are often overprescribed when more selective options are available [12]. Each year over 30 million fluoroquinolone prescriptions are dispensed from outpatient pharmacies in the US. Prior studies have shown that about 5% of these prescriptions are potentially unnecessary and 20% are not recommended as first-line treatment [12]. The use of fluoroquinolones is associated with an increased risk of *Clostridioides difficile* infection, selection of resistance, and several serious and disabling side effects, such as tendinitis, tendon rupture, myasthenia gravis, QT interval prolongation, severe hypoglycemia, and mental health effects relative to other antibiotics [12,13]. Since 2008, the Food and Drug Administration (FDA) has issued several safety warnings about fluoroquinolones; yet, the overuse of fluoroquinolones has persisted [13]. The FDA has specifically advised healthcare professionals that the risk of serious side effects associated with fluoroquinolones generally outweighs the benefits in patients with acute sinusitis, acute bronchitis, and uncomplicated UTIs who have other treatment options [14]. Beyond the risk of serious side effects, there are concerns related to rising rates of fluoroquinolone resistance [12].

Another area of focus for antibiotic stewardship programs is the use of longer than recommended antibiotic courses [15]. Nearly 75% of antibiotic courses given to adults for common outpatient conditions, including pharyngitis, sinusitis, acute otitis media (AOM), community-acquired pneumonia (CAP), skin and soft tissue infection (SSTI), and acute cystitis, exceed the minimum guideline-recommended durations [15]. The median duration of treatment for most of these conditions is ten days, exceeding the guideline recommendations for sinusitis, CAP, and cellulitis of 5–7 days [15]. Longer courses of antibiotics put patients at an avoidable and increased risk of adverse events, resistant infections, and *C. difficile* infection, compared to shorter courses [16,17,18].

This paper focuses on the disparities in inappropriate antibiotic prescribing in patients living in rural areas. There are several reasons why rural-residing patients may be at a higher risk of inappropriate antibiotic prescribing, including decreased access to care and diagnostic testing equipment, such as X-ray, ECG, and biochemistry and microbiology testing facilities [6,9,19]. Clinics in rural areas may have limited hours or days of operation that do not fit with patients’ schedules [20]. Prescribers may opt to empirically prescribe broad-spectrum antibiotics, such as fluoroquinolones, or more extended courses of therapy to avoid additional in-person visits for rural-residing patients who have to drive longer distances to access care [9]. Characterization of antibiotic use by rurality could uncover areas of inappropriate use disproportionally impacting patients living in rural areas. A better understanding of risky antibiotic utilization in rural-residing patients could help inform antibiotic stewardship interventions and lead to more appropriate outpatient antibiotic use. As such, we sought to examine the association of rural residence with suboptimal outpatient antibiotic use in the national Veterans Affairs (VA) Healthcare System.

## 2. Results

We identified 1,405,642 patients who were dispensed an antibiotic in a VA outpatient setting and diagnosed with an upper respiratory tract infection, pneumonia, UTI, or SSTI between 2010 and 2020, of which 22.8% lived in a rural location. Demographics and comorbidities of VA outpatients by rurality are presented in Table 1. As expected, the rural-residing veterans were significantly different (*p* < 0.05) from those residing in urban locations. The rural-residing veterans were older (60.7 vs. 58.3 years) and more likely to be of the White race (83.1% vs. 68.8%) and less likely to be of the Black race (9% vs. 21.7%) and other races (7.9% vs. 9.5%) than the urban-residing veterans. The distribution of rural- versus urban-residing veterans varied by region. Several chronic conditions, including chronic pulmonary disease (19.1% vs. 15.5%), diabetes mellitus (31.3% vs. 28.7%), and hypertension (56.2% vs. 51.1%), were more common in the rural-residing veterans. The median Charlson comorbidity score for the cohort was 0, and the median Elixhauser score was 2.

**Figure 1:**
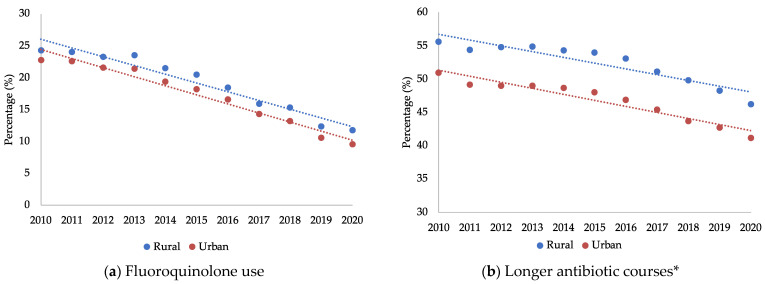
Trends in suboptimal antibiotic use stratified by rurality. * Longer antibiotic courses were defined as prescriptions with durations of ten days or greater.

Trends in suboptimal antibiotic use by rurality are presented in Figure 1a,b. From 2010 to 2020, there was a 9.9% annual decrease in fluoroquinolone use among the rural-residing veterans (95% CI, −11.9% to −7.8%) versus an annual 10.6% decrease among the urban-residing veterans (95% CI, −12.3% to −8.8%). During that time, there was also a 2.1% annual decrease in longer antibiotic courses of therapy among the rural-residing veterans (95% CI, −2.7% to −1.5%) versus a 2.2% annual decrease in the urban-residing veterans (95% CI, −2.8% to −1.7%).

**Figure 2:**
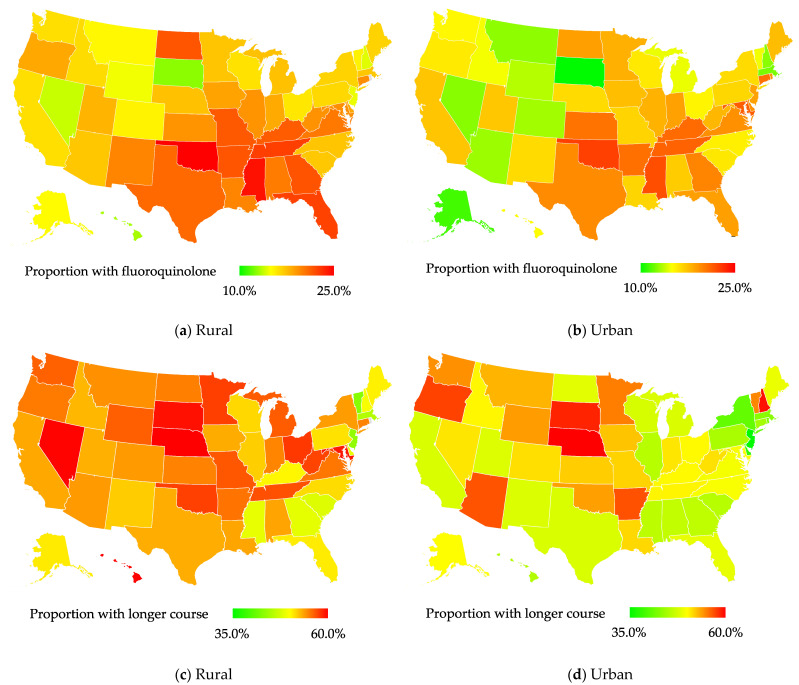
Trends in suboptimal antibiotic use stratified by rurality. Longer antibiotic courses were defined as prescriptions with durations of ten days or greater.

Outpatient antibiotic treatments are presented in Table 1. The rural-residing veterans were more likely to be prescribed fluoroquinolones (19.0% vs. 17.5%) and cephalosporins (12.1% vs. 11.6%) than the urban-residing veterans. In adjusted analyses, the rural-residing veterans were more likely to be prescribed fluoroquinolones (adjusted odds ratio (aOR), 1.03; 95% CI, 1.02–1.04; Table 2), and this was consistent across every region except the South and the West (Supplemental Table S1). Fluoroquinolone use was most common in the South (20.9% and 19.0%) and Midwest (18% and 16.7%) regions for both rural- and urban-residing veterans (Supplemental Table S1, and Figure 2a,b). The results varied by diagnosis. The rural-residing veterans diagnosed with upper respiratory tract infections and SSTIs were more likely to be prescribed fluoroquinolones than the urban-residing veterans, while those diagnosed with UTIs were less likely to be prescribed fluoroquinolones (Table 3).

The median duration of antibiotic prescriptions for our cohort of VA outpatients was 9 days (interquartile range (IQR), 5–10). The median duration was longer in the rural-residing veterans (10 days; IQR, 5–10) than in the urban-residing veterans (8 days; IQR, 5–10). In adjusted analyses, the rural-residing veterans were more likely to be prescribed longer antibiotic courses (aOR, 1.19; 95% CI, 1.18–1.20; Table 2), and this was consistent across every region (Supplemental Table S1). Longer antibiotic courses were most common in the Midwest (55.9% and 50.3%) and the West (54.6% and 50.7%) for both rural-residing and urban-residing veterans (Supplemental Table S1 and Figure 2c,d). The results were consistent across infection diagnosis (Table 3). The adjusted mean antibiotic duration was 0.30 days longer (95% CI, 0.28–0.32) in the rural-residing veterans than in the urban-residing veterans (mean adjusted duration, 7.41 days; 95%, CI 7.35–7.47).

## 3. Discussion

Our work based on over 1.4 million veterans diagnosed with an upper respiratory tract infection, pneumonia, UTI, or SSTI demonstrates that the rural-residing veterans were disproportionally treated with potentially suboptimal outpatient antibiotic prescriptions during our study period from 2010 to 2020. Exposure to longer antibiotic courses was 19% higher in the rural-residing veterans as compared to the urban-residing veterans. Exposure to fluoroquinolones was 3% higher in the rural-residing veterans as compared to the urban-residing veterans. This work identified an important disparity in the treatment of rural-residing veterans and an important process metric and target for antibiotic stewardship programs and other interventions that target antibiotic prescribing.

There are approximately 4.7 million rural-residing veterans in the US, representing 25% of all veterans, and 2.7 million rural-residing veterans are enrolled in VA [21]. Rural residence was previously associated with poor access to care, suboptimal health status, and a higher prevalence of chronic diseases as compared to urban residence [22,23,24]. In VA, the enrolled rural-residing veterans are known to be significantly older, more medically complex, and more likely to have diabetes, obesity, high blood pressure, and heart conditions as compared to the urban-residing veterans [21]. Therefore, our findings of a disparity in higher fluoroquinolone rates and longer prescribing periods in the rural-residing veterans was not entirely unexpected. Previous work, though not specific to antibiotic use, demonstrated that potentially inappropriate outpatient prescribing is more common among rural-residing veterans than urban-residing veterans [19]. This previous cross-sectional study of 1,549,824 older veterans with regular VA primary care and medication use found the rural-residing veterans were at a significantly higher risk of inappropriate prescribing according to all the four quality indicators used (Zhan criteria drugs to avoid, Fick criteria drugs to avoid, therapeutic duplication, and drug–drug interactions) [19]. The authors postulated that their results may have been related to the rural-residing veterans having a diminished access to high-quality specialty care [19]. Our results, too, may have been related to the rural-residing veterans having a decreased access to high-quality infectious diseases (ID) care. Prior work showed that rural-residing veterans with HIV often live more than 60 minutes from ID specialists, and their use of ID care was 17% lower when their travel time to access ID care increased from less than 15 min to over 90 min [25].

We found that the rural-residing veterans were more likely to be prescribed fluoroquinolones and longer antibiotic courses for common outpatient infections, including upper respiratory infections, pneumonia, UTIs, and SSTIs. Suboptimal antibiotic use is dependent on the origin of the infectious disease and the disease severity. Our results related to longer antibiotic courses were consistent across infection diagnosis. However, significant differences in fluoroquinolone prescribing between the rural- and urban-residing veterans varied based on infection diagnosis. The rural-residing veterans were more likely to be prescribed fluoroquinolones than the urban-residing veterans for upper respiratory infections and SSTIs, but less likely for UTIs. Choices to use fluoroquinolone and/or longer antibiotic courses may be more likely to be appropriate for complicated UTIs in our cohort of mostly older males, but less likely for upper respiratory infections and SSTIs.

Our results are consistent with previous studies which suggested associations between antibiotic overuse and rural residence at the individual patient and provider levels [4,5,6,7,8]. An observational cohort study of 670,450 commercially insured women aged 18–44 years in the US treated for uncomplicated UTI found high rates of antibiotic prescriptions for inappropriate agents (46.7%) or durations (76.1%; adjusted risk ratio, 1.10; 95% CI, 1.10–1.10) compared with the urban-residing women [6]. Similarly to our results, this previous study also demonstrated a decline in the proportion of women who received inappropriate agents in both the rural-residing (from 46.6% to 44.8%) and urban-residing women (from 48.8% to 43.5%) over the study period from 2011 to 2015 [6]. The use of inappropriate durations also declined among the urban-residing women (from 77.1% to 72.0%) and less so among the rural-residing women (from 85.1% to 83.2%) [6]. We found that the exposure to longer antibiotic courses decreased similarly in the rural-residing (−2.1%) and urban-residing (−2.2%) veterans, but the use of fluoroquinolones decreased less in the rural-residing veterans (−9.9%) than in the urban-residing (−10.6%) veterans. Our findings of improvements in outpatient antibiotic use over our study period may be related to the increase in antibiotic stewardship efforts throughout VA since the VA National Antimicrobial Stewardship Task Force (ASTF) was chartered in May 2011 [26]. However, despite improvements, additional efforts are needed to improve outpatient antibiotic prescribing in rural-residing veterans.

Our results were consistent across regions, with the rural-residing veterans being more likely to be prescribed fluoroquinolones (except the South and the West) and longer antibiotic courses in all the four regions. Mixed results were found in a previous large national cohort of women with uncomplicated UTI, with the rural-residing women having a varying likelihood of receiving inappropriate agents by region but consistently being more likely to receive an inappropriate duration across all the regions [6]. 

There are several reasons that rural-residing veterans may be exposed to more risky outpatient antibiotic prescriptions beyond remoteness and limited access to high-quality ID care. A recent systematic review identified several factors influencing antimicrobial prescribing in rural and remote primary healthcare settings, related to upstream factors, healthcare facilities, physicians and other healthcare providers, and patients in these settings [27]. While this work was focused on antibiotic use in rural healthcare settings, many of these factors may be applicable to rural-residing patients. For example, physicians and other prescribers may have concerns for patient safety and a low threshold to prescribe antibiotics to rural-residing patients who show signs of a viral infection due to fears of complications arising from a secondary bacterial infection and issues related to the patient being unable to access follow-up care [28,29]. Rural-residing patients may come to expect antibiotics if they drive long distances and put pressure on providers to prescribe antibiotics [27,30]. Rural areas are disadvantaged in accessing health information, and antibiotics are more likely to be given to patients with lower antimicrobial-related knowledge [30,31]. Our results may also be related to institutional racism, which creates structural barriers to access and use of health care among rural non-White patients, and personally mediated racism by rural healthcare providers towards non-White patients [32].

Rural health disparities and antibiotic stewardship are important VA priorities [21,33]. As such, work is needed to identify antibiotic stewardship approaches that can improve the use of outpatient antibiotics in rural-residing VA patients. The use of telehealth may be one approach to improve the use of antibiotics in rural-residing veterans. The Infectious Diseases Society of America (IDSA) supports the use of telehealth technologies to provide up-to-date, timely, cost-effective ID care in rural areas [34]. 

There are limitations to this observational study which utilized secondary data sources. There are important differences (known and unknown) between rural-residing veterans and urban-residing patients who receive suboptimal antibiotics. We did adjust for known and measured confounders of rurality and suboptimal antibiotic use; however, our results may be biased by residual confounding due to other unknown and/or unmeasured factors (such as political factors, institutional and personally mediated racism, and discrimination). Our work is also limited by the accuracy of the data captured by the data sources, particularly pharmacy data. We only captured the antibiotics filled within the VA healthcare system, and therefore our data may underestimate exposure, given that some patients may obtain their antibiotics from external sources (e.g., community outpatient clinics, community hospitals, non-VA community pharmacies). While our population was limited to patients with a recent diagnosis of a common outpatient infection, our results are limited in that indications for the antibiotics prescribed were not captured. We assumed that the infection diagnosis captured represented the antibiotic indication.

Our definition of rural–urban status was based on zip code data rather than distance or access to urbanized areas or VA healthcare facilities. Additionally, we measured fluoroquinolone use and longer courses of antibiotics as surrogates of potentially inappropriate outpatient prescribing practices since there is no gold-standard definition for inappropriate outpatient prescribing. As such, some of the fluoroquinolone prescribing and longer durations may have been appropriate based on best-practice recommendations of the Infectious Diseases Society of America. For example, for community-acquired pneumonia, levofloxacin or moxifloxacin are recommended for outpatients, for pyelonephritis or complicated UTI, fluoroquinolones are recommended, and for group A streptococcal pharyngitis, an antibiotic duration of 10 days is recommended [35,36,37]. Moreover, our definition did not include other elements of suboptimal antibiotic prescribing, such as the drug chosen (beyond fluoroquinolones), dose, and shorter than recommended duration. Due to our large sample size, we may have identified statically significant differences that lack clinical significance. Finally, the generalizability of these results from the VA population to the general US population is limited due to the differences in demographics between the populations, including, age, sex, race, and comorbidity burden.

Future research should continue to develop a consensus definition of inappropriate outpatient antibiotic prescribing and investigate disparities among rural-residing patients. 

## 4. Materials and Methods

We conducted a retrospective study using the national VA data accessed through the VA Informatics and Computing Infrastructure (VINCI) [38]. VA is the most extensive integrated healthcare system in the US, operating over 140 VA medical centers and 1200 outpatient clinics. While VA operates both inpatient and outpatient facilities, we only included the patients that were dispensed antibiotics outside of a hospital admission.

The datasets used included outpatient and inpatient pharmacy data, patient demographics, vital status, inpatient and outpatient medical visits, surgeries, procedures, microbiology results, laboratory results, and vital signs. We included the veterans with an upper respiratory infection, pneumonia, UTI, or SSTI diagnosis and acute outpatient antibiotic dispensing (duration < 30 days) between January 2010 to December 2020. We included the first qualifying outpatient antibiotic treatment course during the study period, which included all the antibiotics dispensed from the start to the end of treatment.

### 4.1. Rurality and Region

We defined rurality using the five-digit US Postal Service zone improvement plan (ZIP) codes of the veteran’s home address per the definitions of the Rural–Urban Commuting Areas (RUCA) [39,40]. We used a common algorithm to collapse RUCA codes into a four-level geographic residence variable (urban, rural-micropolitan, small rural towns, isolated rural towns) [41]. Rural-residing veterans were defined as those living in rural-micropolitan, small rural towns, or isolated rural towns, and urban-residing veterans were defined as those living in urban areas. We used the veteran’s state of residence to determine the region. The regions of residence were divided into four standard census regions: Northeast, Midwest, South, and West [41].

### 4.2. Antibiotic Dispensing

Antibiotic dispensing was evaluated by agent, class, and duration. The following antibiotic classes were evaluated: penicillins (amoxicillin, ampicillin, penicillin), beta-lactams with increased activity (amoxicillin/clavulanate, ampicillin/sulbactam), fluoroquinolones (ciprofloxacin, levofloxacin, moxifloxacin), macrolides (azithromycin, clarithromycin, erythromycin), cephalosporins (cefaclor, cefadroxil, cefazolin, cefotetan, cefoxitin, cefprozil, cefuroxime, cephalexin), tetracyclines (tetracycline, minocycline, doxycycline), and urinary tract antibiotics (sulfamethoxazole/trimethoprim, fosfomycin, nitrofurantoin). Antibiotic class definitions were based on those used by the Centers for Disease Control and Prevention (CDC) for outpatient antibiotic prescriptions [42]. The CDC’s antibiotic class definitions are based on the Uniform System of Classification (USC), a therapeutic classification system created by IQVIA America and the Pharmaceutical Marketing Research Group [43].

### 4.3. Covariates

We evaluated demographics and comorbidities, where comorbidities were identified using the International Classification of Diseases, Ninth or Tenth Revision (ICD-9 or 10) diagnosis and procedure codes in the year prior to the first day of antibiotic treatment, including the summary comorbidity Charlson and Elixhauser scores.

### 4.4. Statistical Analyses

We first assessed rural–urban temporal trends differences in suboptimal outpatient antibiotic use, defined as (1) fluoroquinolone use and (2) longer antibiotic courses, over the study period [15]. Longer courses were defined as prescriptions with durations of ten days or greater. Time trends were assessed with Joinpoint regression to calculate the average annual percent changes (AAPC) and 95% confidence intervals (CI). Significance was set at *p* < 0.05. Additionally, we plotted state-level maps of suboptimal outpatient antibiotic use, stratified by rurality. 

We compared demographics, comorbidities, and antibiotic prescriptions between the rural-residing and urban-residing veterans. We used the chi-squared or Fisher’s exact tests for the categorical variables, where appropriate, and *t*-tests or nonparametric Wilcoxon tests for the continuous variables, where appropriate. To examine the relationship between rurality and suboptimal outpatient antibiotic use, defined as (1) fluoroquinolone use and (2) longer antibiotic courses, we used logistic regression to estimate odds ratios (ORs) and 95% confidence intervals (CIs) adjusting for confounders. Previous work demonstrated that comorbidity burden (including the Charlson score) is associated with potentially inappropriate medications in VA patients [44]. Confounders significantly associated with rurality and suboptimal outpatient antibiotic use were controlled for in the adjusted models (backwards, manual, generalized linear mixed models with a binary distribution and logit link, initial selection *p*-value < 0.1, retained-in-model *p*-value < 0.001) and accounted for clustering by region and year. We used generalized linear models to compare the mean durations of antibiotic courses by rurality. We also conducted stratified analyses by region and infection diagnosis.

## 5. Conclusions

Our large national cohort of over 1.4 million veterans diagnosed with upper respiratory infections, pneumonia, UTIs, and SSTIs demonstrated two areas of risky outpatient prescribing, specifically fluoroquinolone use and longer antibiotic courses, were disproportionally higher among our rural-residing veterans. Fluoroquinolone use and longer antibiotic prescribing are process metrics that should be followed by antibiotic stewardship teams in rural and urban outpatient patients. Telehealth approaches that provide patients with an increased access to high-quality specialized ID care and antibiotic stewardship may be helpful in reducing risky antibiotic prescribing practices in rural-residing patients.

## Figures and Tables

**Table 1:** Demographics, comorbidities, and antibiotic exposures of the Veterans Affairs (VA) outpatients with antibiotic prescriptions in 2010–2020 by rurality.

Demographics, Clinical Characteristics, and Antibiotics	All VA Outpatients (n = 1,405,642)	Rural-Residing Veterans (n = 320,320)	Urban-Residing Veterans (n = 1,085,322)
Age, years, mean (standard deviation)	58.9 (16.5)	**60** **.7 (15.** **7** **)**	**58** **.3 (16.** **7** **)**
Male	1,240,607 (88.3)	**289,301 (90.3)**	**951,306 (87.7)**
Race			
Black	264,286 (18.8)	**28,784 (9)**	**235,502 (21.7)**
White	1,012,373 (72)	**266,080 (83.1)**	**746** **,293 (68** **.8)**
Other	128,983 (9.2)	**25,456 (7.9)**	**103,527 (9.5)**
Hispanic or Latino	80,338 (5.7)	**7555 (2.4)**	**72,783 (6.** **7** **)**
Married	679,007 (48.3)	**174,970 (54** **.6)**	**504,037 (46.4)**
Region of the VA facility			
Northeast	195,292 (13.9)	**35,769 (11.2)**	**159,523 (14.7)**
South	651,218 (46.3)	**150,946 (47.1)**	**500,272 (46.** **1** **)**
Midwest	309,192 (22)	**92,800 (29.0)**	**216,392 (19.** **9** **)**
West	249,940 (17.8)	**40,805 (12.7)**	**209,135 (19.** **3** **)**
Charlson score higher than the median ^a^	689,918 (49.1)	**168,087 (52.5)**	**521,831 (48.1** **)**
Elixhauser score higher than the median ^b^	549,107 (39.1)	**129,796 (40.5)**	**419,311 (38.6)**
Comorbidities			
Cerebrovascular disease	81,640 (5.8)	**19,620 (6.1)**	**62,020 (5.7)**
Alcohol disorder	137,772 (9.8)	**27,779 (8.7)**	**109,993 (10.** **1** **)**
Atherosclerosis	214,987 (15.3)	**57,054 (17.8)**	**157,933 (14.6)**
Cancer or malignancy	315,275 (22.4)	**76,118 (23** **.8)**	**239,157 (22)**
Chronic kidney disease	90,950 (6.5)	20,722 (6.5)	70,228 (6.5)
Chronic pulmonary disease	229,020 (16.3)	**61,205 (19.** **1** **)**	**167,815 (15.5)**
Cognitive disorders	59,077 (4.2)	**12,249 (3.** **8** **)**	**46,828 (4.** **3)**
Congestive heart failure	73,988 (5.3)	**18,219 (5.** **7** **)**	**55,769 (5.1** **)**
Depression	297,322 (21.2)	**66,277 (20.** **7** **)**	**231,045 (21.3)**
Diabetes mellitus	411,669 (29.3)	**100,249 (31.** **3** **)**	**311,420 (28.7)**
Drug abuse	88,609 (6.3)	**15,144 (4.** **7** **)**	**73,465 (6.** **8** **)**
Hypertension	734,535 (52.3)	**180,169 (56.2)**	**554,366 (51.** **1)**
Liver disease	53,231 (3.8)	**10,138 (3.2)**	**43,093 (4)**
Myocardial infarction	29,420 (2.1)	**7535 (2.** **4** **)**	**21,885 (2)**
Obesity	250,600 (17.8)	**59,134 (18.5)**	**191,466 (17.6)**
Peptic ulcer disease	12,602 (0.9)	**3150 (1)**	**9452 (0.9** **)**
Peripheral vascular disease	85,432 (6.1)	**21,473 (6.** **7** **)**	**63,959 (5.9** **)**
Pulmonary heart disease	20,892 (1.5)	4665 (1.5)	16,227 (1.5)
Thyroid disorder	11,1239 (7.9)	**27,646 (8.** **6** **)**	**83,593 (7.** **7** **)**
Infection diagnosis			
Upper respiratory tract infection	782,090 (55.6)	**180,502 (56.4)**	**601,588 (55.4)**
Pneumonia	105,381 (7.5)	**25,847 (8.1)**	**79,534 (7.3)**
Urinary tract infection	222,867 (15.9)	**49,245 (15.4)**	**173,622 (16)**
Skin and soft tissue infection	336,970 (24)	**74,205 (23.2)**	**262,765 (24.2)**
Duration of antibiotics, days, median (IQR)	9 (5–10)	**10 (** **5–** **10)**	**8 (5–10)**
Antibiotic agents *			
Amoxicillin	111,523 (7.9)	**24,788 (7.7)**	**86,735 (8)**
Amoxicillin/clavulanate	251,029 (17.9)	57,457 (17.9)	193,572 (17.8)
Azithromycin	309,658 (22)	**66,898 (20.9)**	**242,760 (22.4)**
Cefuroxime	15,401 (1.1)	**5021 (1.** **6** **)**	**10,380 (1** **)**
Cephalexin	134,703 (9.6)	**30,198 (9.4** **)**	**104,505 (9.6)**
Ciprofloxacin	123,640 (8.8)	**28,703 (9)**	**94,937 (** **8** **.7** **)**
Clindamycin	58,317 (4.1)	**12,550 (3.9** **)**	**45,767 (4.2)**
Doxycycline	121,218 (8.6)	27,360 (8.5)	93,858 (8.6)
Levofloxacin	54,019 (3.8)	**13,277 (4.1)**	**40,742 (3.8)**
Moxifloxacin	74,232 (5.3)	**19,134 (6)**	**55,098 (5.1)**
Nitrofurantoin	22,423 (1.6)	**4796 (1.5)**	**17,627 (1.6)**
Penicillin	12,188 (0.9)	**2133 (0.7** **)**	**10,055 (0** **.9)**
Sulfamethoxazole/trimethoprim	176,881 (12.6)	40,526 (12.7)	136,355 (12.6)
Antibiotic classes *			
Penicillinsc	123,902 (8.8)	**26,982 (8.4)**	**96,920 (8.9)**
Cephalosporins ^d^	150,503 (10.7)	**35,296 (11)**	**115,207 (10.6)**
Macrolides^e^	319,765 (22.8)	**69,451 (21.7)**	**250,314 (23.1)**
Fluoroquinolones ^f^	250,909 (17.9)	**60,890 (19)**	**190,019 (17.5)**
Beta-lactams with increased activity ^g^	251,042 (17.9)	57,463 (17.9)	193,579 (17.8)
Tetracyclines ^h^	128,753 (9.2)	**28,892 (9)**	**99,861 (9.2)**
Urinary tract antibiotics ^i^	198,682 (14.1)	45,173 (14.1)	153,509 (14.1)

The data are n (%) unless otherwise indicated. The categorical variables were compared using the chi-squared or Fisher’s exact tests where appropriate, the means were compared using *t*-tests, and the medians were compared using nonparametric Wilcoxon tests. Bold indicates the *p*-value < 0.05. * Counts and percentages are not mutually exclusive as the patients may have had multiple antibiotic exposures during their treatment course. We included the first outpatient antibiotic treatment course (including all antibiotics from the start to the end of treatment). ^a^ Those with a Charlson comorbidity score higher than the median were compared to those with a median Charlson comorbidity score. The median Charlson comorbidity score was 0. ^b^ Those with a Elixhauser score higher than the median were compared to those with a median Elixhauser score or lower. The median Elixhauser score was 2. ^c^ Penicillins: amoxicillin, ampicillin, penicillin. ^d^ Cephalosporins: cefaclor, cefadroxil, cefazolin, cefotetan, cefoxitin, cefprozil, cefuroxime, cephalexin. ^e^ Macrolides: azithromycin, clarithromycin, erythromycin. ^f^ Fluoroquinolones: ciprofloxacin, levofloxacin, moxifloxacin. ^g^ Beta-lactams with increased activity: amoxicillin/clavulanate, ampicillin/sulbactam. ^h^ Tetracyclines: tetracycline, minocycline, doxycycline. ^i^ Urinary tract antibiotics: sulfamethoxazole/trimethoprim, fosfomycin, nitrofurantoin.

**Table 2:** Association between rural residence and suboptimal antibiotic use.

Suboptimal Antibiotic Use	Rural-Residing Veterans (n = 937,007)	Urban-Residing Veterans (n = 3,002,794)	Unadjusted Odds Ratio (95% Confidence Interval)	Adjusted Odds Ratio (95% Confidence Interval)
Fluoroquinolone exposure ^a^	60,890 (19.0%)	190,019 (17.5%)	**1.11 (1.10–1.12)**	**1.03 (1.02–1.04)**
Longer antibiotic course *^,b^	172,433 (53.8%)	526,423 (48.5%)	**1.24 (1.23–1.25)**	**1.19 (1.18–1.20)**

The data are n (%) or adjusted odds ratio (95% confidence interval). Bold indicates the *p*-value < 0.05 for comparison of the rural and nonrural residence. The adjusted odds ratios were estimated from generalized linear mixed models with a binary distribution and logit link, accounting for clustering by region and year. * Longer antibiotic courses were defined as prescriptions with durations of ten days or greater. ^a^ Adjusted for age, infection diagnosis, cerebrovascular disease, chronic pulmonary disease, depression, hypertension, liver disease, peripheral vascular disease, malignancy, Charlson comorbidity score higher than the median, sex, race, region, and year. ^b^ Adjusted for age, infection diagnosis, atherosclerosis, alcohol disorder, cerebrovascular disease, Elixhauser score higher than the median, depression, hypertension, liver disease, myocardial infarction, obesity, malignancy, Hispanic ethnicity, marital status, sex, race, region, and year.

**Table 3:** Association between rural residence and suboptimal antibiotic use by infection diagnosis.

Suboptimal Antibiotic Use by Infection Diagnosis	Rural-Residing Veterans, n (%)	Urban-Residing Veterans, n (%)	Adjusted Odds Ratio	Lower 95% Confidence Interval	Upper 95% Confidence Interval
Fluoroquinolone exposure ^a^					
Upper respiratory infection	21,876 (12.1%)	60,639 (10.1%)	**1.10**	**1.08**	**1.11**
Pneumonia	12,929 (50%)	39,126 (49.2%)	0.94	0.93	1.02
Urinary tract infection	23,950 (48.6%)	84,841 (48.9%)	**0.92**	**0.90**	**0.94**
Skin and soft tissue infection	5633 (7.6%)	16,801 (6.4%)	**1.12**	**1.09**	**1.16**
Longer antibiotic course *^,b^					
Upper respiratory infection	91,276 (50.6%)	269,462 (44.8%)	**1.21**	**1.20**	**1.22**
Pneumonia	9211 (35.6%)	25,730 (32.4%)	**1.11**	**1.08**	**1.14**
Urinary tract infection	25,796 (52.4%)	78,181 (45%)	**1.23**	**1.20**	**1.25**
Skin and soft tissue infection	50,966 (68.7%)	168,205 (64%)	**1.16**	**1.14**	**1.18**

Bold indicates the *p*-value < 0.05 for the comparison of rural and nonrural residence. The adjusted odds ratios were estimated from generalized linear mixed models with a binary distribution and logit link, accounting for clustering by region and year. * Longer antibiotic courses were defined as prescriptions with durations of ten days or greater. ^a^ Adjusted for age, cerebrovascular disease, chronic pulmonary disease, hypertension, liver disease, peripheral vascular disease, malignancy, Charlson comorbidity score higher than the median, sex, race, region, and year. ^b^ Adjusted for age, atherosclerosis, alcohol disorder, cerebrovascular disease, Elixhauser score higher than the median, depression, hypertension, liver disease, myocardial infarction, obesity, malignancy, Hispanic ethnicity, marital status, sex, race, region, and year.

## Data Availability

The study data may be made available upon reasonable request and approval by the Department of Veterans Affairs.

## Supplementary Materials

The following supporting information can be downloaded at: https://www.mdpi.com/article/10.3390/antibiotics12020224/s1, Table S1: Association between rural residence and suboptimal antibiotic use by region.
