# Supplementary material for: Antibiotic Prescribing in Outpatient Settings: Rural Patients Are More Likely to Receive Fluoroquinolones and Longer Antibiotic Courses
Source: Antibiotics (Basel). 2023 Jan 20;12(2):224. doi: 10.3390/antibiotics12020224 (PMC9952143; doi:10.3390/antibiotics12020224)
Supplement: Supplementary file 1 [file antibiotics-12-00224-s001.zip › antibiotics-2050714-supplementary.pdf]

Supplemental Table S1. Association between rural residence and suboptimal antibiotic use by region.

Clinical outcomes	Rural-residing veterans	Urban-residing veterans	Adjusted odds ratio	Lower confidence interval	Upper confidence interval
Fluoroquinolone exposure[a]					
Northeast	5,860 (16.4%)	26,293 (16.5%)	**1.04**	**1.01**	**1.08**
South	31,588 (20.9%)	95,250 (19.0%)	1.01	0.992	1.03
Midwest	36,040 (16.7%)	16,714 (18.0%)	**1.06**	**1.04**	**1.09**
West	6,728 (16.5%)	32,436 (15.5%)	1.03	0.999	1.07
Longer antibiotic course[b]					
Northeast	18,323 (51.2%)	67,605 (42.4%)	**1.36**	**1.33**	**1.40**
South	79,929 (53%)	243,863 (48.8%)	**1.15**	**1.13**	**1.16**
Midwest	51,908 (55.9%)	108,871 (50.3%)	**1.21**	**1.19**	**1.23**
West	22,273 (54.6%)	106,084 (50.7%)	**1.14**	**1.11**	**1.16**

The data are n (%) or adjusted odds ratio (95% confidence interval). Bold indicates the *p*-value < 0.05 for the comparison of rural and nonrural residence. The adjusted odds ratios were estimated from generalized linear mixed models with a binary distribution and logit link, accounting for clustering by region and year. Longer antibiotic courses were defined as prescriptions with durations of ten days or greater. [a] Adjusted for age, infection diagnosis, cerebrovascular disease, chronic pulmonary disease, hypertension, liver disease, peripheral vascular disease, malignancy, Charlson comorbidity score higher than the median, sex, race, and year. [b] Adjusted for the following: age, infection diagnosis, atherosclerosis, alcohol disorder, cerebrovascular disease, Elixhauser score higher than the median, depression, hypertension, liver disease, myocardial infarction, obesity, malignancy, Hispanic ethnicity, marital status, sex, race, region, and year.
